# Sinonasal symptoms in migraine without aura: results from the cross-sectional ‘Migraine in Poland’ study

**DOI:** 10.3389/fneur.2023.1321261

**Published:** 2023-11-17

**Authors:** Marcin Straburzynski, Magdalena Nowaczewska, Ewa K. Czapinska-Ciepiela, Anna Gryglas-Dworak, Slawomir Budrewicz, Marta Waliszewska-Prosół

**Affiliations:** ^1^Department of Family Medicine and Infectious Diseases, University of Warmia and Mazury, Olsztyn, Poland; ^2^Department of Otolaryngology, Head and Neck Surgery and Laryngological Oncology, Ludwik Rydygier Collegium Medicum in Bydgoszcz, Nicolaus Copernicus University in Toruń, Bydgoszcz, Poland; ^3^Epilepsy and Migraine Treatment Centre, Krakow, Poland; ^4^MIGRE Polish Migraine Center, Wroclaw, Poland; ^5^Department of Neurology, Wroclaw Medical University, Wroclaw, Poland

**Keywords:** headache, rhinorrhea, sinusitis, nose, pain

## Abstract

**Background:**

Migraine without aura (MwoA) is often mistaken for rhinosinusitis. The purpose of this study was to assess the prevalence of sinonasal symptoms, sinusitis-targeting medication use and burden of migraine in a large group of people with MwoA attacks accompanied by rhinologic symptoms.

**Methods:**

Data was collected in a cross-sectional online survey based on an adapted population-based study questionnaire. The analysis included the prevalence of rhinorrhea, mucopurulent nasal discharge, nasal congestion, facial pressure and tenderness to palpation, hyposmia/anosmia and osmophobia.

**Results:**

1,679 (52.73%) MwoA people were identified among 3,225 respondents (women *n* = 2,809, 87.10%) aged 13–80 years (median age 39; standard deviation 10.4). 1004/1679 (59.8%) migraine patients reported one or more rhinologic symptoms and 341/1679 (20.3%) MwoA respondents had symptoms that met rhinosinusitis clinical diagnostic criteria during their headache attacks. In migraine patients, osmophobia was associated with hyposmia [*n* = 141 (12.7%) vs. *n* = 41 (7.2%); *p* = 0.001] and a sensation of unpleasant smells [*n* = 216 (19.4%) vs. *n* = 45 (8.5%); *p* = 0.001], while facial tenderness to palpation was associated with facial allodynia [*n* = 532 (50.4%) vs. *n* = 211 (33.9%); *p* < 0.001]. People with migraine accompanied by rhinosinusitis-like symptoms experienced more disease burden and used ‘sinus medications’ more often.

**Conclusion:**

MwoA patients with rhinosinusitis-like symptoms during migraine attacks require cautious assessment, especially that some symptoms seem to have little value in distinguishing between these disorders (i.e., facial tenderness, hyposmia), while many of these patients have a greater disease burden and therefore often choose medications targeting rhinologic instead of neurologic mechanisms.

## 1 Introduction

Despite different etiology, migraine and rhinosinusitis (RS) are commonly confused with each other; especially migraine symptoms are often misattributed to sinonasal inflammation (1, 2). Firstly, it happens because pain in migraine is often perceived in areas located directly over the paranasal sinuses (i.e., forehead, bridge of the nose, maxillary area) (3). Secondly, migraine headache is often accompanied by nasal cranial autonomic symptoms (CAS), (i.e., nasal congestion and rhinorrhea) (4–6) – a neurogenic response with a complex association with other migraine features (7). Thirdly, there is a significant overlap in seasonal peaks of migraine attacks and RS (8). Finally, the concept of ‘sinus headache’ is universally present in the media, advertising and public awareness. This socio-cultural conditioning directs patients and doctors toward a ‘sinus headache’ diagnosis (9).

The 3rd edition of International Classification of Headache Disorders (ICHD-3) defines headache attributed to acute and chronic RS (10) (Table 1). Both of these sets of criteria require the presence of clinical, nasal endoscopic and/or imaging (e.g., computed tomography – CT) evidence of RS. Moreover, evidence of causation should be demonstrated by the fact that the headache has developed in temporal relation to the onset of RS, or that pain occurs/waxes and resolves/wanes alongside symptoms of RS. Alternatively, causation may be confirmed by headache ipsilaterally to unilateral sinonasal inflammation, or the headache should be exacerbated by pressure applied over the paranasal sinuses (10).

**Table 1:** Relevant *rhinosinusitis* definitions in European Position Paper on Rhinosinusitis and Nasal Polyps 2020 (EPOS) and 3rd edition of International Classification of Headache Disorders (ICHD-3).

**Clinical definition of rhinosinusitis in adults according to EPOS 2020** -inflammation of the nose and the paranasal sinuses characterized by two or more symptoms, one of which should be either nasal blockage / obstruction / congestion or nasal discharge (anterior / posterior nasal drip): •± facial pain/pressure •± reduction or loss of smell and either -endoscopic signs of: ⚬nasal polyps, and/or ⚬mucopurulent discharge primarily from middle meatus and/ or ⚬oedema / mucosal obstruction primarily in middle meatus and/or CT changes: ⚬mucosal changes within the ostiomeatal complex and/or sinuses
**Headache attributed to chronic or recurring rhinosinusitis according to ICHD-3** A.Any headache fulfilling criterion C B.Clinical, nasal endoscopic and/or imaging evidence of current or past infection or other inflammatory process within the paranasal sinuses. C.Evidence of causation demonstrated by at least two of the following: 1.headache has developed in temporal relation to the onset of chronic rhinosinusitis 2.headache waxes and wanes in parallel with the degree of sinus congestion and other symptoms of the chronic rhinosinusitis 3.headache is exacerbated by pressure applied over the paranasal sinuses 4.in the case of a unilateral rhinosinusitis, headache is localized and ipsilateral to it. D.Not better accounted for by another ICHD-3 diagnosis.

The above mentioned classification depends on a correct RS diagnosis. This should be established according to diagnostic criteria provided by, e.g., the European Position Paper on Rhinosinusitis and Nasal Polyps 2020 (EPOS) (11) or the International Consensus Statement on Allergy and Rhinology: Rhinosinusitis 2021 (ICAR-RS) (12). Despite slight differences in disease timeline and type of symptoms required for diagnosis, both sets of these criteria enumerate the following cardinal RS symptoms in adults:

- Nasal blockage/obstruction/congestion.
- Nasal discharge (anterior/posterior).
- Facial pain/pressure.
- Reduction/loss of smell.

As mentioned above, the misdiagnosis of migraine as ‘sinus headache’ is common and has been evaluated by several previous studies (9, 13, 14). Yet, apart from CAS, the broad spectrum of sinonasal symptoms prevalence has so far been analyzed only in studies of people consulted for so called ‘sinus headache’ – many of whom had been eventually diagnosed with migraine or tension-type headache. Therefore, it is unknown how often the general population of migraine patients reports symptoms fulfilling RS clinical diagnostic criteria and what are the points in patients’ history that help to distinguish migraine without aura (MwoA) from RS. Hence the aim of this study is to fill these gaps in scientific data by analyzing sinonasal complaints in a well-defined large group of migraine patients. Symptoms used by EPOS and ICHD-3 classifications have been of special interest to us. We hypothesized that patients with migraine have more prevalent nasal CAS, osmophobia and cacosmia than people with tension-type headache. Moreover, we expected that MwoA and osmophobia patients rarely have hyposmia, which would suggest the usefulness of osmophobia-hyposmia antagonism in distinguishing between migraine and RS. Moreover, we hypothesized that facial tenderness to palpation has a strong overlap with facial allodynia, which would indicate limited value of this symptom in differential diagnosis with RS. Furthermore, this study aims to assess treatment patterns and burden of MwoA accompanied by sinonasal complaints – the hypothesis was that patients with RS-like symptoms in migraine would wait longer for correct diagnosis, and hence use sinus-targeted medications more often.

## 2 Methods

This study is a primary analysis of data collected in the ‘Migraine in Poland’ study - a cross-sectional online survey [the detailed material and methods were recently published (15)]. The study was approved by the Wroclaw Medical University Commission of Bioethics and registered in the ClinicalTrials.gov database (NCT05087420). Data was collected via an online questionnaire from August 2021 till June 2022. The invitation to participate in the study was widely publicized through diverse channels, including national mass media, social media, healthcare providers and large institutions both from the public and private sector. There were no limitations to participate in the study, apart from the fact that respondents could submit their answers only once. Participants received no reimbursement for their contribution and had to provide informed consent before starting the questionnaire. All collected data was anonymized. The submission of the questionnaire by respondents required answering all questions, thus preventing missing data.

The sociodemographic profile, headache symptoms, treatment patterns and burden were assessed with questions from the validated American Migraine Prevalence and Prevention Study (AMPP) (16), adjusted for Poland-specific conditions (e.g., participants were asked about use of medications available in Poland). Comorbidities, including common rhinologic disorders, were assessed using questions about diagnoses confirmed by healthcare professionals. Respondents were also asked about headache abortive and prophylactic medications. Moreover, the study evaluated rhinologic and cranial autonomic symptoms and their temporal relation to headache (i.e., whether these symptoms occurred/waxed and resolved/waned alongside headache). The diagnostic criteria applied in this study included ICHD-3 (for MwoA, migraine aura, cluster headache, episodic tension-type headache (eTTH) and headache attributed to RS) (10) and EPOS 2020 (for RS) (11). Several questions assessed the prevalence of olfactory complaints (anosmia, hyposmia, osmophobia and a sensation of unpleasant smell, i.e., cacosmia). The group of respondents with eTTH without comorbid migraine was used as a comparator for the group studied (MwoA). Migraine-related burden was assessed with the Migraine Disability Assessment (MIDAS) questionnaire.

The statistical analysis adopted the significance level of *p* < 0.05 for the verification of differences between groups. Descriptive statistics included percentages, medians and standard deviations. Tests based on the χ^2^ distribution analyzed variables expressed at ordinal or nominal level. In the case of 2×2 tables, the continuity correction was applied. However, Fisher’s exact test was used for tables larger than 2×2, when the conditions for using the χ^2^ test were not met. A non-parametric test (Mann–Whitney U) analyzed continuous variables broken down into groups. Calculations were made in the statistical environment R ver.3.6.0, PSPP program and MS Office 2019. Minimal sample size of n = 385 MwoA participants for 95% confidence level was calculated with Fisher’s exact test.

## 3 Results

3,225 respondents (87.1% women) aged 13–80 years (median age 39; standard deviation 10.4) submitted complete questionnaires in this study. From these respondents, 1,679 (52.73%) participants fulfilled ICHD-3 diagnostic criteria for MwoA. eTTH without comorbid MwoA, typical migraine aura or cluster headache was present in 210 (6.5%) respondents. The remaining 1,336 respondents did not fulfill all ICHD-3 diagnostic criteria for MwoA or TTH. Thanks to the broad distribution of the invitation to participate in the study (national and social media), the response rate is roughly estimated to be 0.1%.

### 3.1 Sinonasal symptoms in MwoA

**Figure 1:**
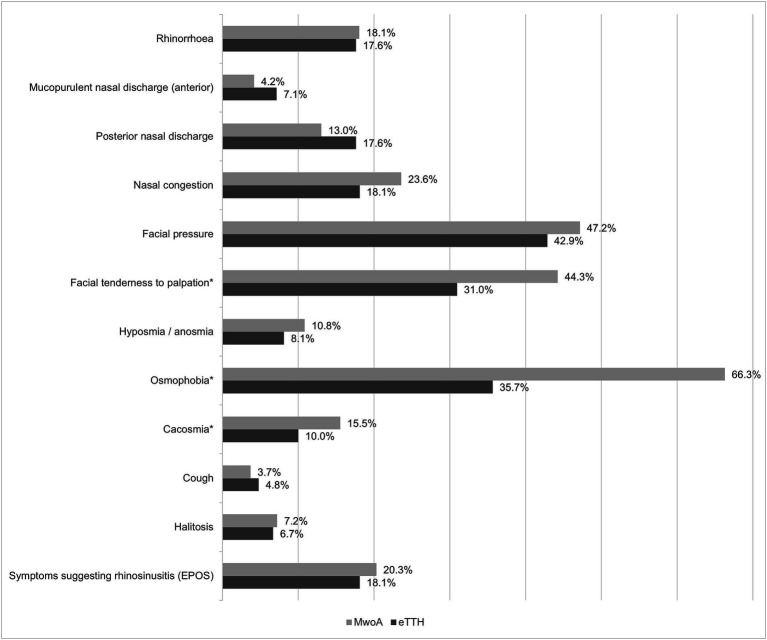
Prevalence of sinonasal symptoms during headache attack among patients with migraine without aura (MwoA) and episodic tension-type headache (eTTH).

MwoA respondents had at least one sinonasal symptom during migraine attacks in 1004 (59.8%) cases, while in 744 (44.31%) at least one rhinologic complaint was also present in the interictal phase. The prevalence of sinonasal symptoms is presented in Table 1 and CAS in Table 2. Patients with MwoA experienced a significantly (χ^2^ = 50.8; degrees of freedom (df) = 12; *p* = 0.001) higher prevalence of osmophobia, perception of an unpleasant smell [i.e., cacosmia (17)] and facial tenderness during headache than the eTTH patients (Figure 1). However, the prevalence of other sinonasal symptoms was not significantly different between MwoA and eTTH respondents. Further analysis showed that osmophobia was strongly associated with cacosmia [*n* = 216 (19.4%) vs. *n* = 45 (8.5%); *p* = 0.001] and hyposmia [*n* = 141 (12.7%) vs. *n* = 41 (7.2%); *p* = 0.001] reported by MwoA patients. Moreover, MwoA respondents with hyposmia significantly more often reported nasal congestion than patients without it: *n* = 81 (44.51%) vs. *n* = 101 (7.9%); *p* < 0.001. Facial tenderness to palpation was associated with facial allodynia among MwoA respondents [*n* = 532 (50.4%) vs. *n* = 211 (33.9%); *p* < 0.001]. Patients with RS-like symptoms during headache had allodynia more often than other MwoA subjects [*n* = 248 (78.7%) vs. *n* = 948 (69.5%); *p* = 0.001] (see Table 3).

**Table 2:** Prevalence of sinonasal symptoms in MwoA.

Symptom	Symptom present in headache phase *n* = 1,679 (%)	Location in respect to pain *n* (%)	Symptom present in headache-free phase *n* = 1,679 (%)
Clear/watery nasal discharge (anterior) (rhinorrhea)	303 (18.1)	Bi. 194 (11.6) Ipsi. 101 (6.0) Con. 8 (0.5)	367 (21.7)
Mucopurulent nasal discharge (anterior)	70 (4.2)	Bi. 58 (3.5) Ipsi. 9 (0.5) Con. 3 (0.2)	77 (4.6)
Nasal discharge (posterior)	219 (13.0)	NA	356 (21.2)
Nasal congestion	396 (23.6)	Bi. 241 (14.4) Ipsi. 149 (8.9) Con. 6 (0.4)	343 (20.4)
Facial pressure	792 (47.2)	Bi. 195 (11.6) Ipsi. 581 (34.6) Con. 16 (1.0)	269 (16.0)
Facial tenderness to palpation	743 (44.3)	Bi. 255 (15.2) Ipsi. 474 (28.2) Con. 14 (0.8)	235 (14.0)
Hyposmia / anosmia	182 (10.8)	NA	120 (7.2)
Osmophobia	1,113 (66.3)	NA	310 (18.5)
RS symptoms fulfilling clinical EPOS 2020 diagnostic criteria	341 (20.3)	NA	213 (12.7)

Bi – bilateral, Ipsi. – ipsilateral, Con. – contralateral, NA- not applicable, RS – rhinosinusitis, EPOS - European Position Paper on Rhinosinusitis and Nasal Polyps 2020.

**Table 3:** Interictal non-nasal cranial autonomic symptoms (CAS) in respondents with migraine without aura (MwoA).

Symptom	*n* = 1,679 (%)	Location in respect to pain *n* = 1,679 (%)
Lacrimation	648 (38.6)	Bil. 348 (20.7) Ipsi. 277 (16.5) Con. 23 (1.4)
Conjunctival injection	397 (23.7)	Bil. 248 (14.8) Ipsi. 137 (8.2) Con. 12 (0.7)
Myosis	269 (16.0)	Bil. 157 (9.4) Ipsi. 94 (5.6) Con. 18 (1.1)
Ptosis	406 (24.2)	Bil. 145 (8.6) Ipsi. 242 (14.4) Con. 19 (1.1)
Facial flushing/sweating	345 (20.6)	Bil. 263 (15.7) Ipsi. 69 (4.1) Con. 13 (0.8)
At least one non-nasal CAS	947 (56.4)	–
At least one CAS	(63.0)	–

Bi – bilateral, Ipsi. – ipsilateral, Con. – contralateral.

MwoA participants reported at least one episode of what they interpreted as ‘sinus headache’ in the previous 12 months in 602 (35.9%) cases and in 520 (31.0%) in the 3 months prior to study. There was no statistically significant difference between MwoA and eTTH groups in respect to the prevalence of ‘sinus headache’ (*n* = 602 (35.9%) vs. *n* = 83 (39.5%); *p* = 0.334).

**Figure 2:**
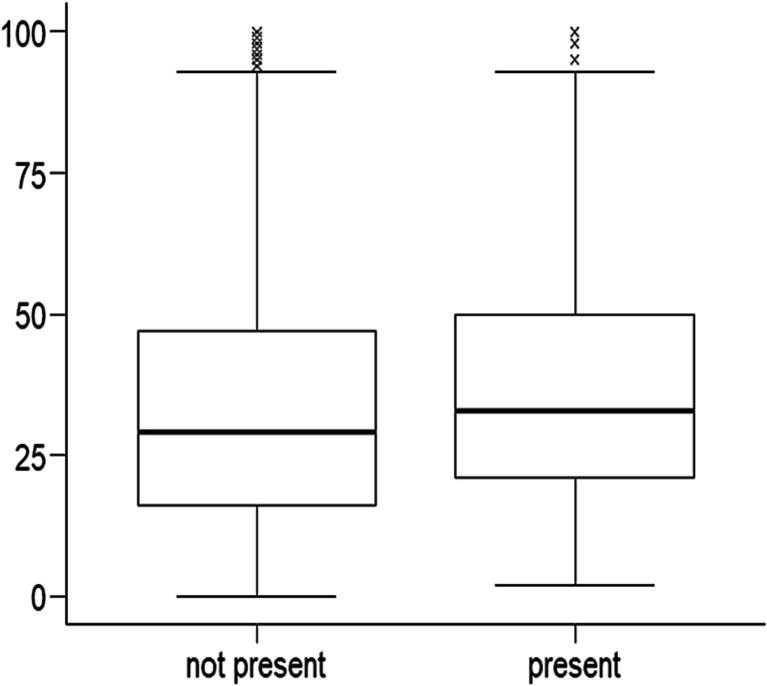
Association between migraine burden expressed on vertical axis as Migraine Disability Assessment (MIDAS) score and presence of EPOS symptoms during headache.

Headache burden, as expressed by Migraine Disability Assessment (MIDAS) score, was significantly higher in MwoA patients with RS-like symptoms (Median = 33 vs. 29; *p* = 0.004) (Figure 2). Despite that, patients with migraine and sinonasal symptoms had similar time from MwoA onset to diagnosis as people without sinonasal symptoms (χ^2^ = 0.741; df = 3; *p* = 0.863). Neither was chronic migraine more prevalent in this group (n = 25 (7.9%) vs. *n* = 92 (6.7%); *p* = 0.531).

### 3.2 Rhinosinusitis diagnostic criteria

The symptoms fulfilled EPOS 2020 clinical diagnostic criteria in 341 (20.3%) of 1,679 MwoA participants; in the majority of cases (*n* = 315) these symptoms occurred episodically, reflecting acute RS diagnosis. 251 (73.6%) participants reported non-nasal CAS in migraine headache, co-occurring with EPOS 2020 symptoms.

ICHD-3 diagnostic criteria for headache attributed to acute RS present in MwoA respondents:

- Criteria C1 and C2-315 (18.8%) respondents experienced symptoms reflecting a RS clinical diagnosis, recurring exclusively during headache attacks;

- Criterion C3-206 (12.3%) respondents described exacerbation of headache by pressure applied over the paranasal sinuses;

- Criterion C4 – nasal symptoms were ipsilateral to unilateral headache in 200 (11.9%) participants.

Overall, criteria B and C were present in 345 (20.6%) MwoA respondents.

### 3.3 Treatment and healthcare utilization

480 (28.6%) of 1,679 MwoA patients consulted with otorhinolaryngologists in the past because of their headache. Furthermore, 316 (18.8%) MwoA patients received a ‘sinus headache’ diagnosis from a physician, although not necessarily from an otorhinolaryngologist. Other rhinologic diagnoses in MwoA group included chronic sinusitis [*n* = 235 (14.0%)], chronic rhinitis [*n* = 623 (37.1%)] and allergy/atopy/allergic rhinitis [*n* = 602 (35.9%). Finally, 244 (14.5%)] MwoA respondents took ‘sinus medications’ to treat their headache at least on 1 day per month [1–4 days: *n* = 187 (11.1%); 5–9 days: *n* = 39 (2.3%); 10 or more: *n* = 18 (1.1%)]. Patients with RS-symptoms during their headache took these medications significantly more often (22.6% vs. 12.5%; χ^2^ = 22.3; df = 2; *p* < 0,001).

## 4 Discussion

To our knowledge, this is the first comprehensive description of sinonasal symptoms in a large population of MwoA patients. We have shown that more than half of MwoA patients report one or more rhinologic symptoms, and one fifth fulfil (at least clinically) diagnostic criteria for RS during their headache attacks. In other words, our results point out that acute RS (mis)diagnosis is initially justified in 20.3% of MwoA patients.

In most of our respondents RS-like symptoms occurred alongside migraine attacks and subsided with headache after 4–72 h. In these situations acute RS can be suspected due to the symptoms’ duration. However, it should be underlined that recurrent acute RS is considered rare and warrants rhinologic evaluation i.e., nasal endoscopic examination and/or CT of the paranasal sinuses (11). This in turn may be challenging to perform interictally, due to the short duration of symptoms. Even if a patient was assessed during a migraine attack, the endoscopic and presumably imaging results can show mucosal edema further contributing to misdiagnosis (18).

Also the presence of chronic (> 12 weeks) sinonasal symptoms does not necessarily confirm a RS diagnosis. In our research 12.7% respondents had RS-like symptoms independently of their headache. This result corresponds with population-based studies, where 11–14% of Europeans report symptoms of chronic RS (19). However, these estimations were verified by population-based imaging studies, which put the prevalence of chronic RS at around 3% in general population (20, 21). The remainder of symptomatic patients most often have rhinitis, especially allergic one. It is estimated that 28.9–36.1% of the Polish population have (mostly allergic) rhinitis (22). The latter diagnosis was also given by healthcare professional to 35% of MwoA participants in our study. This would also explain why a large proportion of respondents confirmed sinonasal and cranial autonomic symptoms in headache-free periods. However, it should be noted that our analysis included subjects in whom these symptoms occurred and disappeared alongside headache.

CAS are one of the main explanations given to justify misdiagnosing migraine as RS (9). In our group these symptoms were highly prevalent, reflecting findings from other studies (4, 5, 23, 24). Nasal congestion and rhinorrhea can contribute to misdiagnosis, as they are also part of diagnostic criteria for RS and rhinitis (11). However, non-nasal CAS are to a lesser extent a manifestation of sinonasal inflammation (with exception of lacrimation and conjunctival injection). Meanwhile, non-nasal CAS are reported to accompany headache in more than half of MwoA patients. In contrast, we have previously shown in patients with coronavirus disease 2019 (COVID-19) that non-nasal CAS accompany acute viral RS in only 13.8% (25). This may suggest that the presence of non-nasal CAS can indicate MwoA, when differential diagnosis with RS is considered. Future studies in this area are required. As a side note, recent reports have shown that the presence of CAS is associated with better response to treatments targeting calcitonin gene related peptide (CGRP) (26, 27). In this context, RS-like symptoms in migraine may have clinical significance as they may help to identify patients with better prognosis for antiCGRP therapies. Finally, nasal CAS in our study were not significantly more prevalent among MwoA respondents when compared to TTH. Considering lack of available data on prevalence of CAS in TTH this suggests a need of future studies in that regard.

This study analyzed olfactory complaints of MwoA subjects in particular. On the one hand, osmophobia is common in migraine (28) and rare in acute RS (25). On the other hand, hyposmia is highly prevalent in RS (11). What is more, osmophobia and hyposmia can be expected to be mutually exclusive - the person complaining of hypersensitivity to smells may be expected not to have decreased olfactory function at the same time. Consequently, the pair of these symptoms could be especially useful in the differentiation between migraine (osmophobia) and RS (hyposmia). In fact, hypersensitivity to smells was a common symptom during migraine attacks in our group (66.3%). However, patients complaining of osmophobia more often reported hyposmia than patients without hypersensitivity to smells. This observation has its confirmation in studies that tested olfactory function in migraine, where osmophobia was often accompanied by hyposmia (28). The association between migraine, osmophobia and hyposmia might be related to structural changes in the olfactory system in patients with migraine (29). Moreover, hyposmia during migraine attack might be related to CAS, and particularly to nasal congestion – our results and other studies have shown that decreased nasal patency is associated with olfactory disfunction (30). Additionally, cacosmia might be in fact a secondary symptom – the unpleasant smell perceived by respondents might be an emanation of general hypersensitivity to smells during migraine attacks. In fact, our results indicate that cacosmia is associated with osmophobia in MwoA. In conclusion, this part of our results points out that the presence of hyposmia is a poor candidate for distinguishing between RS and migraine, due to its higher prevalence in MwoA patients. The role of osmophobia in excluding RS gives more promise, as our previous study has shown that it is a rare symptom in acute viral RS (25) – especially that osmophobia seems to be highly specific to migraine in our results and studies from other groups 31.

ICHD-3 criteria for headache attributed to acute or chronic rhinosinusitis suggests that headache is often exacerbated by pressure applied over the paranasal sinuses. However, there is little scientific data supporting this observation (32) and our previous report shows moderate (22%) prevalence of this symptom in COVID-19-related acute RS. Our present results indicate that facial tenderness occurs in 12.3% of MwoA patients and is strongly associated with facial allodynia. Moreover, facial allodynia was associated with RS-like symptoms in MwoA respondents. It seems therefore that the exacerbation of headache by pressure applied over the paranasal sinuses may be mistaken for allodynia in migraine, and consequently shows little potential as a differentiating symptom when RS and MwoA are considered.

According to earlier studies, migraine patients with so called ‘sinus headache’ more often have its chronic form (33). In our study however we found no such association, although respondents with RS-like complaints had significantly higher burden of the disease. The latter observation reflects findings from other studies, with patients diagnosed with ‘sinus headache’ (34, 35).

Previous studies have shown that almost all patients with migraine misdiagnosed as ‘sinus headache’ have been at least once unnecessarily treated with antibiotics (36, 37), and in many cases undergone sinonasal surgery (34). In our study, 14.5% people with MwoA take medications labelled as ‘sinus’ to treat their headache on regular basis, but this percentage increases to 22.6% if RS-like symptoms accompany migraine attacks. The study by Eross et al. indicates that people with migraine take ‘sinus’ medications due to misconceptions regarding the etiology of complaints. However, it must be remembered that most of these products contain pharmaceuticals also effective in migraine (i.e., paracetamol, non-steroidal anti-inflammatory drugs, caffeine). Moreover, in Poland medications in this group mostly contain pseudoephedrine with ibuprofen or paracetamol. Future studies should investigate the effectiveness of sympathomimetics in people with migraine and sinonasal symptoms, especially that there are is evidence that pseudoephedrine may reduce headache in RS (38) and CGRP levels in migraine with sinonasal symptoms (39).

This study has several limitations, most of which have been addressed in the leading publication describing used methods (15). The major issue lies in convenience sampling, although this has been somehow mitigated by the broad distribution of the survey. The quality of data is furthermore supported by the similarities in our results and population-based studies from our region. Nevertheless, it should be underlined that even the most scrupulous studies with convenience sampling cannot reach the reliability of census studies. Similarly to other cross-sectional studies, this research does not allow for assessment of causality. Moreover, our study is limited by the lack of confirmation of sinonasal symptoms in clinical setting. Consequently, data on endoscopic or imaging findings in our respondents is absent. It is a limitation impossible to avoid in an online survey. Nevertheless, it should be remembered that imaging studies and nasal endoscopic examinations could have helped in identifying subjects with true sinonasal disease. However, an educated guess indicates that only a small proportion of the studied group would have findings typical for RS on CT (20). Moreover, some of these might be related to mucosal edema and discharge observed during migraine attacks. Consequently, future prospective longitudinal studies in a clinical setting are required.

## 5 Conclusion

Symptoms that may mimic acute rhinosinusitis accompany headache in one fifth of people with MwoA. However, this study questions the value of some of patients complaints in distinguishing between migraine and RS (i.e., hyposmia, exacerbation of headache by pressure applied over paranasal sinuses). In uncertain cases, migraine diagnosis may be supported by interictal osmophobia and non-nasal CAS in addition to the symptoms listed in MwoA ICHD-3 diagnostic criteria. However, the symptoms alone are not specific enough and require imaging and/or endoscopic evaluation to exclude rhinologic diseases. The latter may prove challenging in clinical practice, considering that in the majority of cases sinonasal symptoms occur only during migraine attacks. Future studies should concentrate on the prospective clinical assessment of these phenomena, especially that the need for such research is supported by the fact that patients with MwoA and sinonasal symptoms have a greater disease burden and often choose medications targeting rhinologic instead of neurologic mechanisms.

## Data availability statement

The raw data supporting the conclusions of this article will be made available by the authors, without undue reservation.

## Ethics statement

The studies involving humans were approved by Wroclaw Medical University Commission of Bioethics. The studies were conducted in accordance with the local legislation and institutional requirements. Written informed consent for participation in this study was provided by the participants’ legal guardians/next of kin.

## Author contributions

MS: Conceptualization, Data curation, Formal analysis, Investigation, Methodology, Project administration, Resources, Software, Supervision, Visualization, Writing – original draft, Writing – review & editing. MN: Investigation, Resources, Writing – review & editing. EC-C: Investigation, Resources, Writing – review & editing. AG-D: Investigation, Resources, Writing – review & editing. SB: Resources, Supervision, Writing – review & editing. MW-P: Conceptualization, Data curation, Formal analysis, Investigation, Methodology, Project administration, Resources, Software, Supervision, Writing – review & editing.
